# Liver B Cells Promotes MASLD Progression via the Apelin/APLNR System

**DOI:** 10.7150/ijms.101492

**Published:** 2025-01-01

**Authors:** Su Jiang, Jiaxue Lu, Nan Li, Xueqi Bai, Lei Shi, Ziying Tian, Jieyu Zhou, Wenling Zhang

**Affiliations:** 1 Department of Medical Laboratory Science, The Third Xiangya Hospital, Central South University, Changsha, Hunan, China.; 2 Department of Pathology, The second Xiangya Hospital, Central South University, Changsha, Hunan, China.

**Keywords:** apelin/APLNR, metabolic dysfunction-associated steatotic liver disease, B cells

## Abstract

**Aims:** Investigate the role of the apelin/APLNR axis in metabolic dysfunction-associated steatotic liver disease (MASLD), focusing on the progression from metabolic dysfunction-associated simple steatotic liver (MASS) to metabolic dysfunction-associated steatohepatitis (MASH) and fibrosis, with emphasis on liver B cells.

**Methods:** Serum samples from MASLD patients and liver tissues from hepatocellular carcinoma patients were collected to measure apelin and APLNR protein expression. C57BL/6J mouse models of varying MASLD stages were developed using a high-fat diet and CCl_4_. RNA sequencing was used to study the apelin/APLNR axis's regulatory functions in the Raji B cell line.

**Results:** Bioinformatic and clinical analyses show that apelin and APLNR are up-regulated in MASLD, correlating with disease severity. Animal models demonstrate that apelin and ML221 injections affect liver steatosis, inflammation, and fibrosis. Sequencing and RT-PCR in Raji cells indicate that the apelin/APLNR axis promotes the expression of inflammatory cytokines and extracellular matrix molecules.

**Conclusion:** The apelin/APLNR axis is crucial in MASLD progression. Targeting this axis may offer therapeutic potential to modulate B cell function and mitigate MASLD advancement.

## Introduction

Metabolic dysfunction-associated steatotic liver disease (MASLD), previously known as non-alcoholic fatty liver disease (NAFLD), has become one of the most common chronic liver diseases worldwide. With the rapid rise in the global obese population, the incidence and prevalence of MASLD are continuously increasing 1. MASLD is a chronic metabolic disease characterized by hepatic lipid deposition and nonspecific chronic inflammation. Its development involves multiple factors, including genetic, environmental, immune factors, metabolic disorders, and intestinal microbial imbalance 2. Studies have shown that MASLD development is linked to various physiological and pathological processes, such as hepatic lipid metabolism disorders, defects in fatty acid β-oxidation, oxidative stress, inflammatory responses, and dysregulated apoptosis 3. MASLD is considered the hepatic manifestation of metabolic syndrome and is highly associated with metabolic comorbidities, including obesity, type 2 diabetes, hyperlipidemia, and hypertension 4. MASLD is a progressive disease, encompassing metabolic associated steatosis (MASS) and metabolic dysfunction-associated steatohepatitis (MASH) 5. MASS is characterized by simple hepatic steatosis without inflammation, whereas MASH exhibits lobular inflammation, hepatocyte apoptosis, and other more severe pathological features 5. Severe MASLD is often accompanied by irreversible liver fibrosis and can progress to cirrhosis and even hepatocellular carcinoma, significantly impacting patients' quality of life and health 6. Current pharmacological treatments for MASLD face significant challenges, and effective therapeutic drugs are still lacking 7. Therefore, it is crucial to explore the factors and precise mechanisms influencing MASLD progression, especially from MASS and mild MASH to MASLD-related fibrosis. Timely treatment at the MASS and mild MASH stages to delay or reverse MASLD is especially important.

APLNR, also known as APJ, is a member of the G protein-coupled receptor (GPCR) family with seven transmembrane domains 8. Apelin, or APLN, is the endogenous ligand for APLNR, with its C-terminal serving as the specific binding site for the APLNR receptor 8. During signal transduction, apelin activates APLNR, leading to the coupling of APLNR with G proteins and the activation of downstream signaling pathways 9. In the liver, apelin is primarily secreted by endothelial cells, particularly those lining the portal and central veins, as well as by Kupffer cells, which are the resident macrophages 10. Apelin can be processed into various subtypes of different lengths, such as Apelin-12, Apelin-13, Apelin-17, and Apelin-36, retaining their biologically active C-terminus 11, 12. Increasing evidence indicates that apelin/APLNR plays a crucial regulatory role in metabolic diseases. For instance, apelin regulates the ratio of sodium-glucose cotransporter 1 (SGLT-1) to glucose transporter 2 (GLUT2) by promoting AMPK phosphorylation, thereby enhancing glucose transport from the intestinal lumen to the bloodstream 13. Apelin can also activate the Wnt signaling pathway, stimulating the expression of the anti-adipogenic factor Wnt10b, which in turn inhibits the expression of PPARγ and C/EBP, reducing adipogenesis 14. On the other hand, apelin/APLNR suppresses the cAMP/PKA pathway by coupling with Gi proteins, inhibiting lipolysis, and leading to increased intracellular lipid deposition 15. Studies on obese children have shown that serum apelin-12 levels are significantly higher in those with metabolic syndrome 16. A meta-analysis revealed that apelin levels are higher in patients with type 2 diabetes compared to controls, but plasma apelin concentrations are lower in newly diagnosed and untreated type 2 diabetes patients than in healthy controls 17. These findings suggest that apelin/APLNR may be closely related to the occurrence and development of MASLD. However, there have not been any studies clearly reporting the specific roles and mechanisms of apelin/APLNR in the progression of MASLD.

During the development of MASLD, the inflammatory environment is primarily controlled by immune cells. Recent evidence supports the involvement of B cells in MASLD progression. B cells can produce immunoglobulins, present antigens, and secrete cytokines after being activated by pathogen-associated molecular patterns through Toll-like receptors, affecting immune-mediated inflammatory responses in various ways 18. B cells accumulate in the livers of patients with MASH, who exhibit high levels of lobular inflammation and fibrosis, suggesting that B cells may alter the disease course 19. In mouse models of MASH, B cells have pro-inflammatory roles involving adaptive immune mechanisms mediated by B cell receptors and innate immune mechanisms dependent on MYD88 20. Additionally, MASH severity is reduced in B cell-deficient mice, with decreased inflammation and fibrosis 20. Apelin/APLNR is involved in the regulation of various immune processes. Co-culture experiments of head and neck cancer cells and macrophages demonstrate that when the expression of apelin in cancer cells is suppressed, the gene expression of macrophage L1β, IL6, and TNFα significantly increases, while the levels of anti-inflammatory cytokines significantly decrease 21. In the nervous system, apelin-13 attenuates LPS-induced inflammation in BV-2 microglial cells by promoting autophagy and enhancing M2 polarization of microglia 22. Conversely, another study revealed that apelin exacerbates osteoarthritis by enhancing the expression of IL-1β through the inhibition of miR-144-3p in synovial fibroblasts via the PI3K and ERK pathways 23.

Here, we demonstrate the changes in apelin and APLNR levels in the context of MASLD. We identify the expression of APLNR in liver B cells in MASLD and validate this finding in a series of MASLD mouse models. This study aims to investigate the molecular mechanism of apelin/APLNR in regulating liver B cells and MASLD progression, addressing a critical gap in understanding and potentially informing therapeutic strategies.

## Materials and Methods

Details are provided in Supplementary information.

### Bioinformatic analysis of public data

RNA sequencing data from the TCGA-LIHC cohort were downloaded and processed using the “TCGAbiolinks” R package. Hallmark gene sets were obtained from the GSEA-MSigDB website, and gene set enrichment analysis (GSEA) was conducted using the “clusterProfiler” R package.

### Cell culture

HepG2 and AML 12 cells were cultured in DMEM/high glucose medium (HyClone) supplemented with 10% fetal bovine serum (Procell) and 1% penicillin/streptomycin (HyClone). Raji cells were cultured in RPMI 1640 medium modified (HyClone) supplemented 10% fetal bovine serum (Procell) and 1% penicillin/streptomycin (HyClone). All cells were cultured in an environment of 37°C and 5% CO_2_.

### Oil red O staining

To pre-treat and stain cells with an Oil Red O staining kit (Beyotime, cat. no. C0157S), aspirate the cell culture medium, and wash with PBS. Fix the cells with 4% paraformaldehyde for 10 minutes, followed by PBS washes. For cryosections of mouse liver tissue, thaw pre-prepared frozen sections from -20 °C for 5-10 minutes. Mix Oil Red O solution with its diluent at a 3:2 ratio, let it sit for 10 minutes, and filter it. Cover the cells with staining wash solution for 20 seconds, remove it, and then stain with the Oil Red O solution for 15 minutes. After removing the stain, cover with staining wash solution for 30 seconds, rinse with PBS for 20 seconds, and counterstain the nuclei with hematoxylin. Finally, cover with PBS for microscopic observation and photography.

### Statistical analysis

The data were presented as mean with standard error of the mean (s.e.m.). Unless indicated otherwise, multiple comparisons among more than two groups were performed using one-way ANOVA with Tukey's multiple comparisons test. Two-group comparisons were performed using either a two-tailed unpaired Student's t-test, a two-tailed unpaired Welch's t-test, a Mann-Whitney U test, or a two-tailed paired Student's t-test, depending on the specific circumstances. All statistical analyses were conducted using GraphPad Prism software version 9 for Mac OS (GraphPad Software). A significance level of *P* < 0.05 was considered statistically significant for all types of analyses.

## Results

### Apelin and APLNR expression were up-regulated in livers of MASLD patients

**Figure 1:**
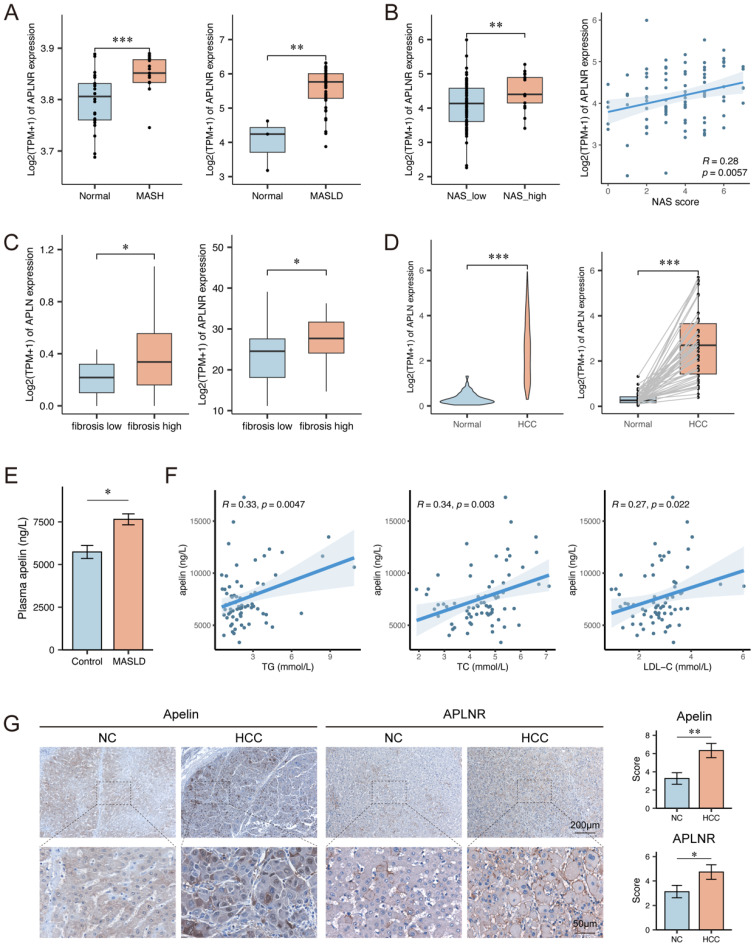
** Apelin and APLNR expression were up-regulated in livers of MASLD and HCC patients.** (A) Box plots showing *APLNR* expression in the GSE89632 and GSE185051 datasets. (B) NAS score and expression of *APLNR* in GSE225740 dataset. (C) Expression of *APLN* and *APLNR* in the GSE240729 dataset among the low liver fibrosis (fibrosis score<2) and high liver fibrosis groups. (D) Unpaired and paired expression of *APLN* in the TCGA-LIHC dataset. (E) The concentration of apelin in the serum of MASLD patients (n=74) and healthy individuals (n=14) measured by ELISA. (F) Correlation analysis between serum apelin concentration and serum TG, TC, and LDL-C in MASLD patients (n=74). (G) Representative IHC staining results of apelin and APLNR in human HCC and peritumor tissues. The statistical analysis of the staining scores is shown on the right (n=30). The P values are calculated by two-tailed unpaired Welch's t-test (A-C, E), Mann-Whitney U test (D, G), two-tailed paired Student's t-test (D), or Spearman's correlation (B, F). *P < 0.05; **P < 0.01; ***P < 0.001

To investigate the expression of APLNR in the liver during MASLD, we performed bioinformatics analysis using datasets GSE89632 and GSE185051. The analysis results from these datasets revealed that the expression levels of *APLNR* in livers of MASLD patients were higher compared to normal individuals (Fig. 1A). Further analysis of the GSE225740 dataset found that MASLD patients with higher NAFLD Activity Score (NAS) had higher hepatic *APLNR* expression levels (Fig. 1B). The analysis results of the GSE240729 dataset indicate that the expression of *APLN* and *APLNR* is higher in the livers of MASLD patients with high fibrosis scores (Fig. 1C). Additionally, data analysis from The Cancer Genome Atlas (TCGA) indicated that hepatocellular carcinoma (HCC) tissues had higher APLN expression compared to adjacent non-tumor tissues (Fig. 1D).

We collected serum samples from MASLD patients (n=74) and normal individuals (n=14) and measured the apelin content in the serum samples by ELISA. The result showed that serum apelin levels were higher in MASLD patients (Fig. 1E). Clinical data analysis revealed a positive correlation between serum apelin levels and serum total triglycerides (TG), total cholesterol (TC), and low-density lipoprotein cholesterol (LDL-C) levels in these 74 MASLD patients (Fig. 1F). Moreover, we collected liver tissue sections from HCC patients (n=30), including both cancerous and adjacent non-cancerous tissues. Immunohistochemical assays were performed to measure apelin and APLNR expression levels, and the results indicated that HCC tissues had higher levels of apelin and APLNR (Fig. 1G). In summary, the expression of apelin and APLNR is up-regulated in the livers of MASLD patients and may increase with the progression of MASLD.

### Apelin and APLNR expression were up-regulated in livers of HFD mice

**Figure 2:**
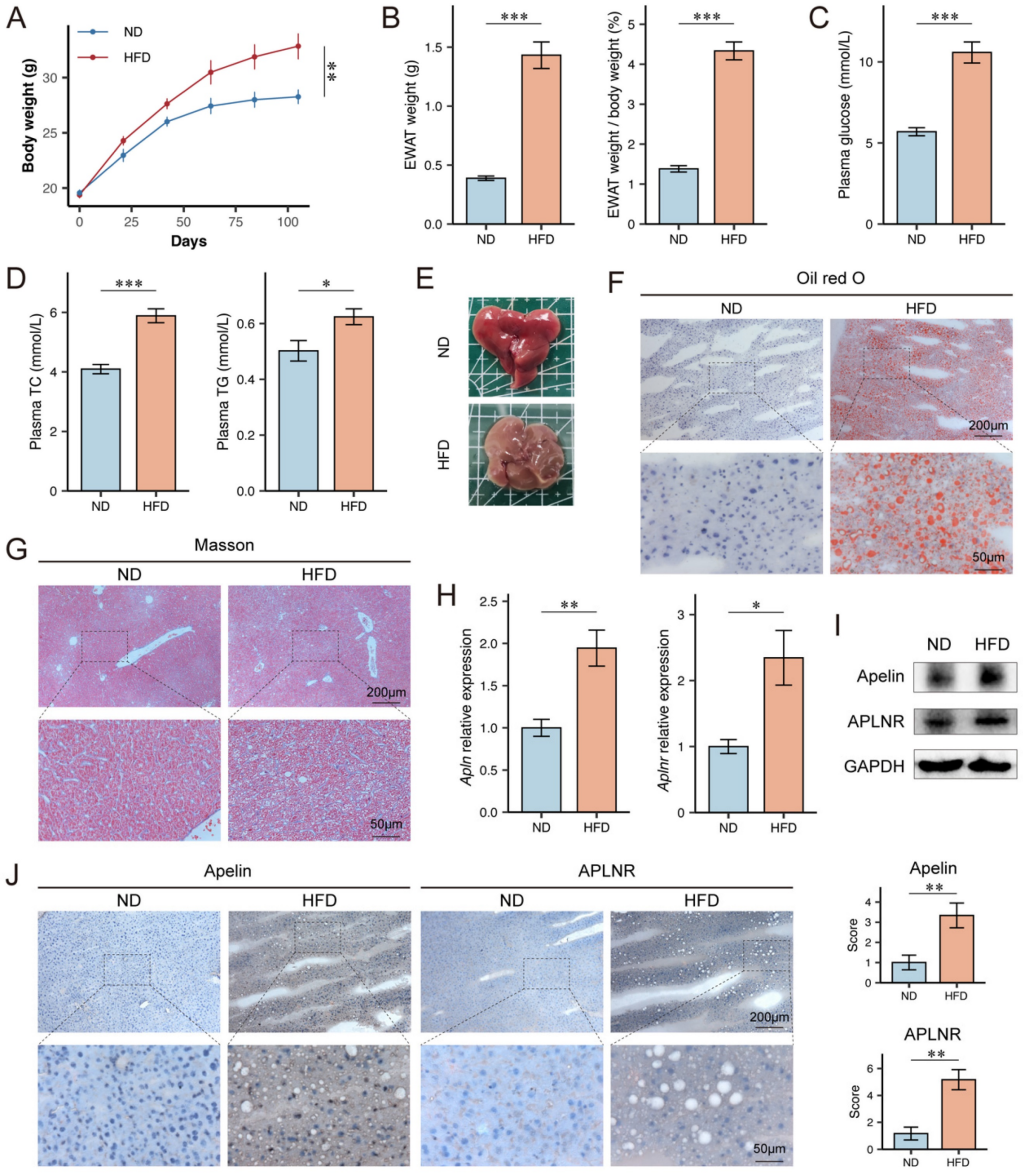
** Apelin and APLNR expression were up-regulated in livers of HFD mice.** (A) Body weight of C57BL/6J mice on ND diet for 15 weeks and HFD diet for 15 weeks, recorded every 3 weeks (n=6). (B) After 15 weeks of feeding, the bilateral epididymal white adipose tissue (EWAT) of the mice was isolated and weighed (n=6). (C-D) Blood was collected from the mouse eye, and serum was separated for the measurement of glucose, TC, and TG using assay kits (n=6). (E) Mouse livers were isolated and photographed. (F) Liver tissues were cryosectioned and stained with an Oil Red O staining kit to visualize and image lipid content under a microscope. (G) Paraffin-embedded sections of the liver tissue were prepared and stained using a Masson's trichrome staining kit to assess the level of fibrosis. (H) RNA was extracted from mouse liver tissues using the Trizol method, and RT-PCR was conducted to determine mRNA levels of *Apln* and *Aplnr* (n=3). (I) Total protein was extracted from mouse liver tissues, and Western blot was used to determine the expression of apelin and APLNR (n=6). (J) Immunohistochemistry was performed on liver cryosections to detect apelin and APLNR. The statistical analysis of the staining scores is shown on the right (n=6). The P values are calculated by two-tailed unpaired Student's t-test (A, C, D), two-tailed unpaired Welch's t-test (B, H), Mann-Whitney U test (J). *P < 0.05; **P < 0.01; ***P < 0.001

To explore the expression levels of apelin/APLNR in MASLD mice, we utilized a high-fat diet (HFD)-induced obesity mouse model, which disrupts the balance between lipid synthesis and degradation, leading to lipid accumulation and cytotoxicity in hepatocytes. Six-week-old male C57BL/6J mice were fed either a normal diet (ND) or an HFD for 15 weeks. Throughout the experiment, the body weight of HFD-fed obese mice was significantly higher than that of ND-fed control mice (Fig. 2A). After 15 weeks of feeding, the weight of epididymal white adipose tissue (EWAT) and its proportion of body weight were significantly higher in HFD-fed mice (Fig. 2B). HFD-fed mice also had higher serum glucose, TC, and TG levels compared to ND-fed mice (Fig. 2C-D). Upon examination of mouse livers, it was observed that HFD-fed mice showed obvious disease characteristics such as a yellowish color and rougher texture compared to ND-fed mice (Fig. 2E). Interestingly, the liver size of HFD-fed mice was not as enlarged as expected when compared to the control group. Weighing the livers showed that HFD-fed mice had lower liver weights than the control group (Fig. S1A-B). We speculate that the abundant fat deposits in the mice's bodies might have compressed the space available for liver growth.

The Oil Red O staining of frozen liver sections from the mice indicated a significant lipid accumulation in the hepatocytes of HFD-fed mice (Fig. 2F). Masson's trichrome staining of paraffin-embedded liver sections indicated that HFD-fed mice had more pronounced collagen fiber deposition, suggestive of increased fibrosis (Fig. 2G). Further analysis of mouse livers revealed higher mRNA expression levels of *Apln* and *Aplnr* in HFD-fed mice (Fig. 2H). Additionally, the liver tissues from HFD-fed mice also exhibited higher protein expression levels of apelin and APLNR (Fig. 2I-J, S1C).

### Establishment of MASLD mouse models at different stages

**Figure 3:**
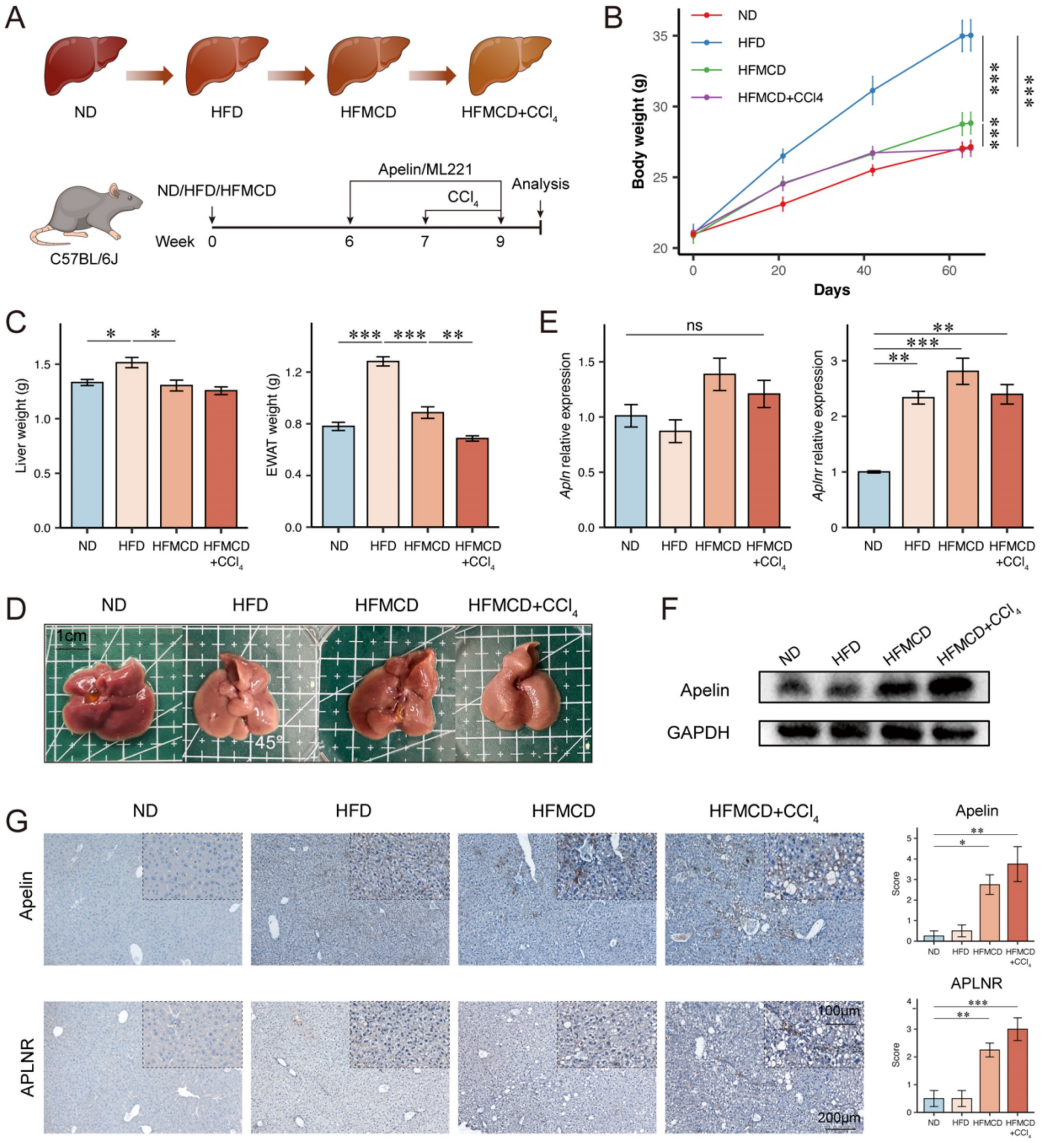
** The expression of apelin and APLNR increased with the progression of MASLD.** (A) Schematic diagram of the establishment of mouse models for different stages of MASLD (n=4). (B) Body weight of ND, HFD, HFMCD, and HFMCD+CCl_4_ groups of mice after 9 weeks of feeding (n=4). (C) After 9 weeks of feeding, the liver and EWAT were isolated from the mice and weighed (n=4). (D) Images of the liver from each group of mice. (E) RNA was extracted from livers of each group, and RT-PCR was used to determine the mRNA expression of *Apln* and *Aplnr* (n=4). (F) Total protein was extracted from each group's mouse liver, and Western blot was used to determine the expression of apelin protein (n=4). (G) Paraffin-embedded liver sections were used for IHC detection of apelin and APLNR. The statistical analysis of the staining scores is shown on the right (n=4). The P values are calculated by one-way ANOVA followed by Tukey's multiple comparisons tests (B-C, E, G). *P < 0.05; **P < 0.01; ***P < 0.001; ns represents no significance

Since we aim to explore the relationship between apelin/APLNR and the progression of MASLD, we tried to construct three types of mouse models to simulate different stages of MASLD. Eight-week-old male C57BL/6J mice were fed an ND as a control, an HFD for 9 weeks to simulate MASS, a choline-deficient high-fat diet (HFMCD) for 9 weeks to simulate MASH, and HFMCD with intraperitoneal injections of CCl_4_ to simulate MASLD-associated liver fibrosis (Fig. 3A). Throughout the experiment, the body weight of HFD-fed obese mice was significantly higher than that of ND-fed control mice. The body weight of HFMCD-fed mice was slightly higher than ND-fed mice, and HFMCD-fed mice experienced weight loss after CCl_4_ injections, likely due to severe liver disease causing malnutrition (Fig. 3B).

Upon dissection of mouse livers, it was observed that compared to the control group, the livers of HFD-fed mice were lighter in color, more yellowish, and heavier. The livers of HFMCD and HFMCD+CCl_4_ groups showed more pronounced disease characteristics with rougher textures and no increase in liver weight (Fig. 3C-D). RT-PCR analysis indicates that while *Apln* mRNA levels remain constant, *Aplnr* mRNA expression is significantly up-regulated in the livers of both the HFD and HFMCD groups (Fig. 3E). At the protein level, Western blot (Fig. 3F, S1D) and immunohistochemistry (Fig. 3G) further demonstrate an increase in apelin and APLNR in the liver, particularly within the HFMCD and HFMCD+CCl_4_ groups. Taken together, these findings revealed that the expression levels of apelin and APLNR in the liver increased to some degree as the disease progressed.

### Apelin promoted the progression of MASLD in mice

**Figure 4:**
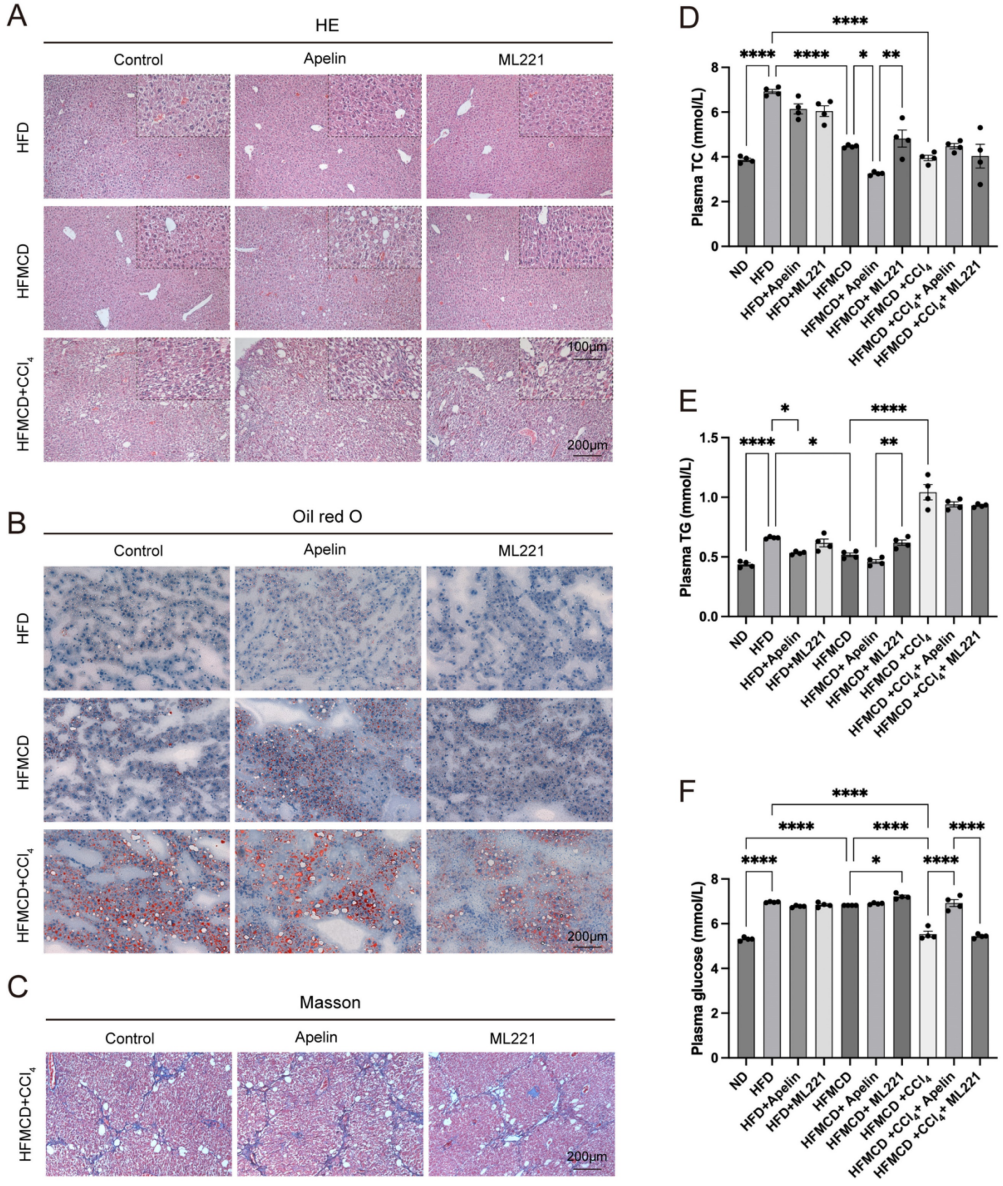
** Apelin/APLNR exacerbated hepatic lipid accumulation and fibrosis in MASLD mice.** (A) HE staining of liver paraffin sections shows pathological changes in the liver after intraperitoneal injection of apelin or ML221. (B) Oil Red O staining of mouse liver frozen sections. (C) Masson's trichrome staining of mouse liver paraffin sections. (D-F) Blood was collected from each group of mice after 9 weeks of feeding, and serum was separated for the measurement of TC, TG, and glucose using assay kits (n=4). The P values are calculated by one-way ANOVA followed by Tukey's multiple comparisons tests (D-F). *P < 0.05; **P < 0.01; ***P < 0.001

To further investigate the impact of apelin/APLNR on MASLD, we conducted intraperitoneal injections of apelin and the APLNR antagonist ML221 in the above-mentioned MASLD mouse models (Fig. 3A). In mice fed an HFD for 9 weeks, hepatocytes exhibited mild steatosis. Due to the relatively mild condition of hepatocyte lesions, the effects of apelin and ML221 were not very apparent. However, in mice fed an HFMCD for 9 weeks, more noticeable pathological changes were observed in the hepatocytes. These changes were exacerbated after apelin injection and alleviated after ML221 injection. Mice fed an HFMCD and injected with CCl_4_ showed significant pathological liver changes (Fig. 4A, S1E).

Oil Red O staining of liver frozen sections demonstrated that hepatic lipid accumulation increased in HFMCD-fed mice after apelin injection (Fig. 4B, S1F). Masson's trichrome staining of liver tissues further indicated that fibrosis increased in the HFMCD+CCl_4_ group following apelin injection and decreased in the group treated with ML221 (Fig. 4C, S1G). In summary, these results suggest that apelin exacerbates liver disease in MASH mice. By measuring the concentrations of TC, TG, and glucose in the serum of mice, it was found that intraperitoneal injection of apelin or ML221 had a certain impact on lipid and glucose metabolism in mice (Fig. 4D-F). However, the specific effects and mechanisms require further investigation.

### Apelin/APLNR might regulate MASLD progression by modulating hepatic B cells

To understand the potential mechanism by which apelin/APLNR affects the progression of MASLD, we first investigated the direct effects of apelin and APLNR on hepatic lipid accumulation. We established a lipid accumulation cell model by adding oleic acid (OA) and palmitic acid (PA) in a 2:1 ratio to the culture medium of HepG2 and AML 12 cells 24. Based on the Oil Red O staining results and the CCK8 cell viability assay, we determined the conditions to be 300 mmol/L OA and 150 mmol/L PA, cultured for 24 hours (Fig. S2A-C). Following the addition of apelin to the cell culture medium, we found that various concentrations of apelin could not alter the lipid accumulation in HepG2 cells caused by OA and PA (Fig. S2D). Similarly, we increased the expression of APLNR in HepG2 and AML 12 cell lines via plasmid transfection (Fig. S3A), but this did not significantly affect the number of intracellular lipid droplets or the levels of intracellular TC and TG (Fig. S3C-F). Furthermore, using siRNA to suppress the expression of APLNR also did not result in any changes of TC and TG levels (Fig. S3B, G-H). Therefore, we hypothesize that apelin/APLNR does not influence the progression of MASLD through the direct regulation of hepatic lipid metabolism.

**Figure 5:**
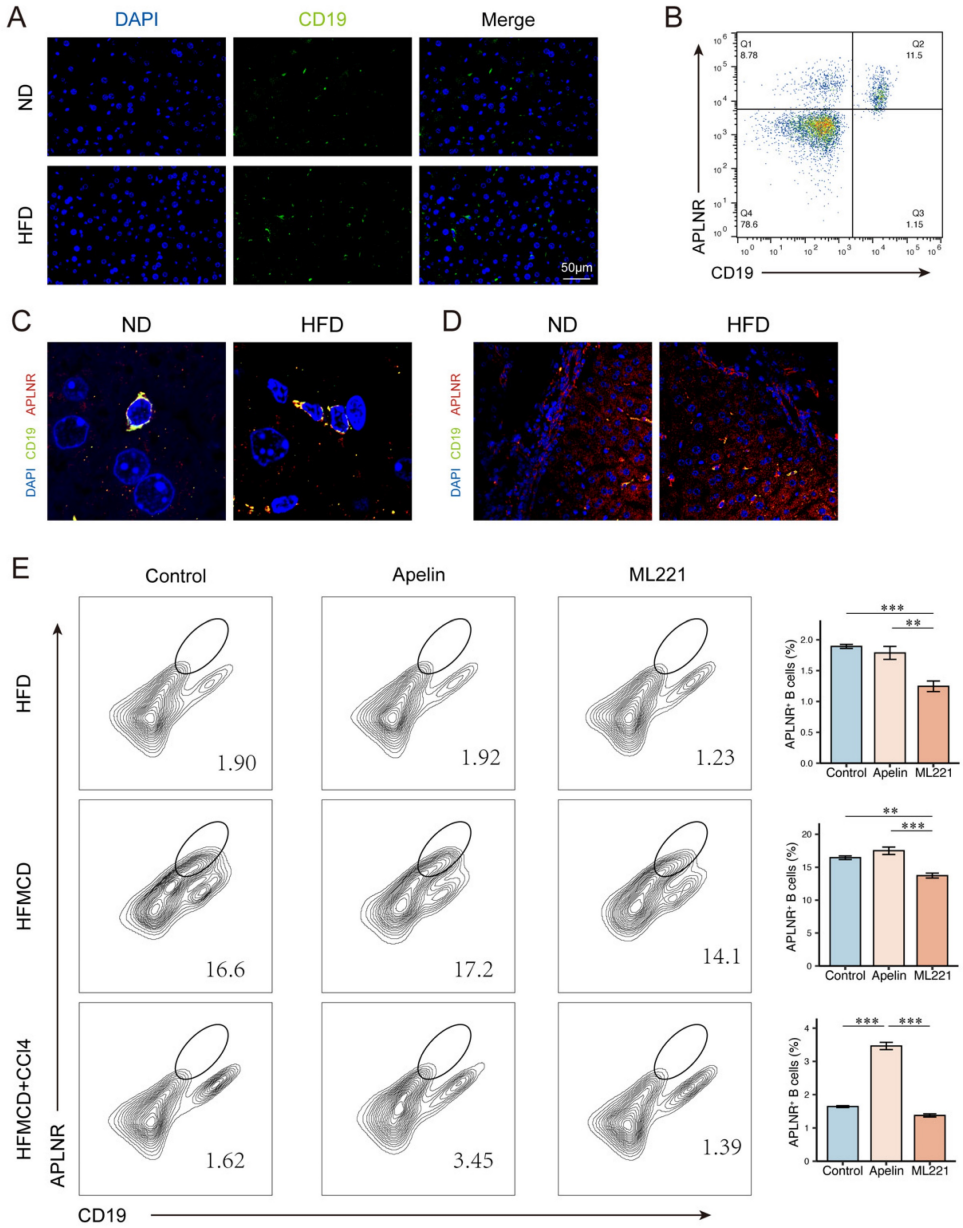
** Apelin/APLNR affected the progression of MASLD by regulating liver B cells.** (A) Immunofluorescence detection of CD19^+^ B cells in liver paraffin sections of C57BL/6J mice fed with HFD for 15 weeks and controls. (B) Flow cytometry analysis of CD19^+^ and APLNR^+^ cells in human peripheral blood PBMCs. (C-D) Immunofluorescence detection of CD19 and APLNR in livers of mice fed with HFD and ND for 15 weeks. (E) After intraperitoneal injection of apelin, ML221, or saline, single-cell suspensions of livers from different model mice were prepared, and immune cells were isolated and analyzed by flow cytometry for CD45, CD19, and APLNR. The P values are calculated by one-way ANOVA followed by Tukey's multi comparison test (E). *P < 0.05; **P < 0.01; ***P < 0.001

In mice fed an HFD for 15 weeks, there was an increase in B cell infiltration in the liver compared to ND-fed mice (Fig. 5A). We collected human peripheral blood and isolated peripheral blood mononuclear cells (PBMCs). Flow cytometry revealed the expression of APLNR on the surface of CD19^+^ B cells (Fig. 5B). Immunofluorescence of mouse liver sections showed that APLNR was also expressed on the surface of B cells within the liver (Fig. 5C-D). Similarly, we also assessed the expression of APLNR in other liver immune cells using immunofluorescence and found that there was minimal expression of APLNR on F4/80^+^ hepatic macrophages and CD8α^+^ T cells (Fig. S4A-B). Flow cytometry of single-cell suspensions from mouse liver tissues indicated that, with apelin injection, the number of CD45^+^ infiltrating hepatic cells increased, including an increase in CD19^+^APLNR^+^ cells (Fig. 5E). In summary, we speculate that apelin/APLNR may exacerbate MASLD by modulating liver B cells.

### Apelin/APLNR enhanced B cell migration ability and promoted the expression of immune molecules

To understand the impact of apelin/APLNR on B cell function, we conducted further studies using Raji cell line, a B-cell lymphoma model that serves as a valuable tool for B cell research 25. APLNR can be successfully overexpressed in Raji cells and the IC_50_ value of apelin is 207.9 μmol/L (Fig. S5A-B).

**Figure 6:**
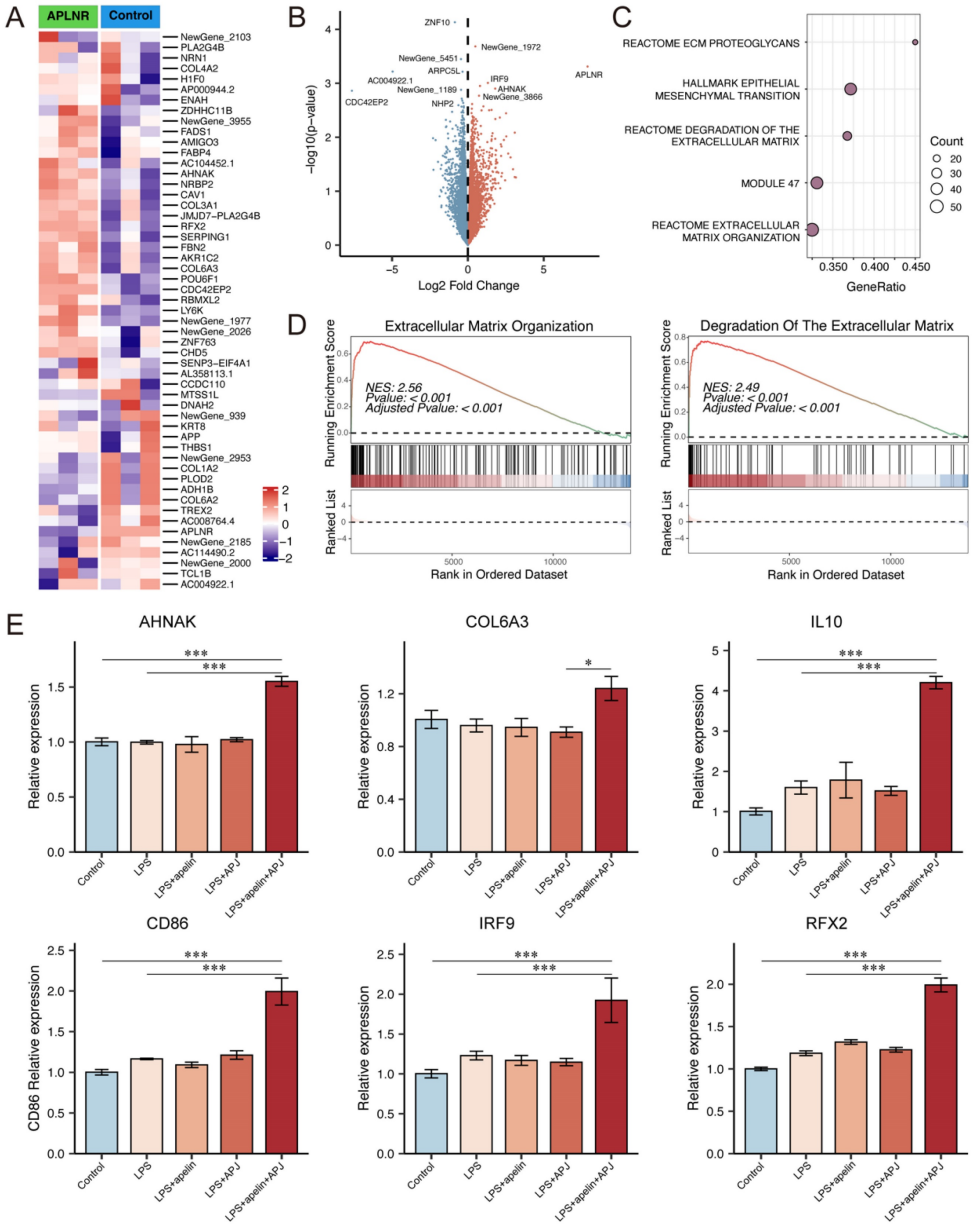
** Apelin/APLNR could promote B cell migration and activation.** (A-B) Cultured Raji cells were transfected with a plasmid to overexpress *APLNR* and were treated with apelin recombinant protein in the culture medium. Transcriptome sequencing was conducted along with the control group (n=3). The heatmap and volcano plot of differentially expressed genes are shown. (C) Dot plot of GSEA pathway analysis. (D) GSEA pathway analysis showing extracellular matrix organization pathway and degradation of the extracellular matrix pathway. (E) After overexpressing *APLNR* in Raji cells through plasmid transfection and adding LPS or apelin to the culture medium, cells were collected for RNA extraction, and RT-PCR was performed to measure the mRNA levels of *AHNAK*, *COL6A3*, *IL10*, *CD86*, *IRF9*, and *RFX2*. The P values are calculated by one-way ANOVA followed by Tukey's multi comparison test (E). *P < 0.05; **P < 0.01; ***P < 0.001

Next, we performed transcriptomic sequencing on APLNR-overexpressing Raji cells supplemented with 5μmol/L apelin, comparing them with a control group. The results showed that genes such as *AHNAK*, *COL6A3*, *COL3A1*, and *IRF9* were up-regulated in cells with high expression of apelin and APLNR (Fig. 6A-B). GSEA pathway analysis results indicated that apelin/APLNR activated extracellular matrix-related pathways (Fig. 6C-D, S5C-E). RT-PCR results demonstrated that apelin/APLNR promotes the expression of molecules such as *AHNAK*, *COL6A3*, *IL10*, *IRF9*, and *RFX2* (Fig. 6E, S5F). In addition, we used western blot analysis to evaluate the activation of signaling pathways and found that the expression of p-PI3K, p-mTOR, p-MAPK, and CyclinD1 was up-regulated in Raji cells following APLNR overexpression (Fig. S5G). These results suggest that apelin/APLNR regulates B cell migration and cytokine expression, thereby promoting MASLD progression through multiple aspects.

## Discussion

As a lipid-regulating factor, apelin has been found to be up-regulated in various diseases, including diabetes and MASLD 16, 26. Apelin is secreted by various cells, including cardiac cells, vascular endothelial cells, adipocytes, and intestinal epithelial cells, and is expressed in multiple organs such as the heart, blood vessels, adipose tissue, and liver 27-29. The apelin secreted by these tissues and organs collectively maintains the levels of apelin in the serum, making it a potential biomarker for metabolism-related diseases. For instance, studies have indicated that serum apelin-12 levels can serve as a useful biomarker for predicting metabolic syndrome in obese children 16. Additionally, the receptor APLNR is present on the cell surface, and its ligand apelin, as well as its antagonist ML221, hold potential as therapeutic agents. Therefore, exploring the specific roles and mechanisms of the apelin/APLNR axis in diseases is of high value.

Some researchers have investigated the role of apelin/APLNR in MASLD, but previous studies have not reached consistent conclusions, and there are significant differences in perspectives among researchers. Some studies suggest that apelin may benefit MASLD patients and help alleviate symptoms, while others indicate that apelin might exacerbate certain aspects of the condition. For instance, systemic apelin treatment has been shown to reduce steatosis in insulin-resistant mice, but this effect is not directly mediated by targeting hepatocytes 30. Conversely, another study found that both *in vivo* and *in vitro* assays indicated a key role for apelin in promoting liver fibrosis, potentially through the ERK signaling pathway in LX-2 cells, leading to the expression of profibrotic genes 31. LX-2 cells are a human hepatic stellate cell (HSC) line that has been established as an important tool for studying the mechanisms of liver fibrosis 32. Given that MASLD is a chronic, relatively slow-progressing metabolic disease with multiple stages, we suspect that differences in pathological features at various stages of MASLD might contribute to these discrepancies in research findings. Therefore, our study focused on the role of apelin at different stages of MASLD. Notably, the addition of CCl_4_ to the dietary regimen is not an ideal model of severe MASLD, as liver fibrosis induced by CCl_4_ may not accurately mimic the fibrosis caused by the progression of MASLD.

Although this study ultimately focuses on B cells to explore the role and potential mechanism of apelin/APLNR, it is important to note that apelin and APLNR are expressed in various liver cells, with their effects on MASLD being collectively regulated by these cells. Apelin and APLNR are expressed in endothelial cells and are involved in regulating their functions. Previous studies have found that the apelin/APLNR system acts as a pro-angiogenic and pro-fibrotic mediator in the liver, with the APLNR antagonist F13A reducing collagen content, improving MAP and PP, enhancing cell viability, and reducing angiogenesis and cell infiltration in fibrotic rat livers 26. Compared to normal livers, APLN protein and gene expression are overexpressed in cirrhotic livers, increasing with the progression of cirrhosis. Particularly in end-stage cirrhosis, APLN is strongly expressed in proliferating arterial capillaries directly connected to sinusoidal structures, suggesting a role for APLN in the proliferation of these capillaries 33.

Apelin and APLNR can also be expressed in hepatic stellate cells and macrophages, affecting liver fibrosis in MASLD. Studies have suggested that, in cholestatic liver injury, apelin signaling exacerbates liver damage and fibrosis, contributing to the progression of cholestasis 34. Apelin has been shown to increase the synthesis of collagen I and platelet-derived growth factor receptor β (PDGFRβ) in LX-2 cells, indicating that apelin may be an important mediator of fibrosis in human liver disease 35. Activation of the apelin/APLNR system may suppress the production of adhesion molecules and inflammatory mediators in RAW264.7 cells (macrophages) 36. Moreover, apelin/APLNR may influence disease severity by regulating T cell functions. A study on the combined use of dendritic cell vaccines and the apelin receptor antagonist ML221 in modulating Th1 and Th2 cell responses in breast cancer mice showed that the combined treatment significantly increased the ratio of Th1 cells while decreasing the ratio of Th2 cells in the spleen compared to controls, and it also reduced serum IL-10 levels 37.

Regarding the direct regulatory relationship between apelin/APLNR and lipid metabolism in cells, existing studies have reached various conclusions. A study on ovarian cancer indicated that apelin derived from adipocytes activates APLNR-expressing tumor cells in a paracrine manner, enhancing lipid uptake and utilization, thus providing energy for the survival of ovarian cancer cells at metastatic sites 38. Activation of the apelin-APLNR pathway in ovarian cancer cells led to increased lipid droplet accumulation, which could be reversed by the addition of F13A or APLNR knockdown 38. However, another research showed that in both Hep3B human hepatoma cells and primary mouse hepatocytes, the apelin/APLNR signaling pathway prevents lipid accumulation and combats hepatic steatosis by activating AMPK and inducing PPARα 39.

In summary, apelin and APLNR are expressed in various cells and play highly complex roles in the progression of MASLD. This study provides an important supplement to the research on the relationship between B cells and MASLD.

## Supplementary Material

Supplementary methods, figures and tables.
