# Supplementary material for: Liver B Cells Promotes MASLD Progression via the Apelin/APLNR System
Source: Int J Med Sci. 2025 Jan 1;22(1):197–208. doi: 10.7150/ijms.101492 (PMC11659828; doi:10.7150/ijms.101492)
Supplement: Supplementary file 1 — Supplementary methods, figures and tables. [file ijmsv22p0197s1.pdf]

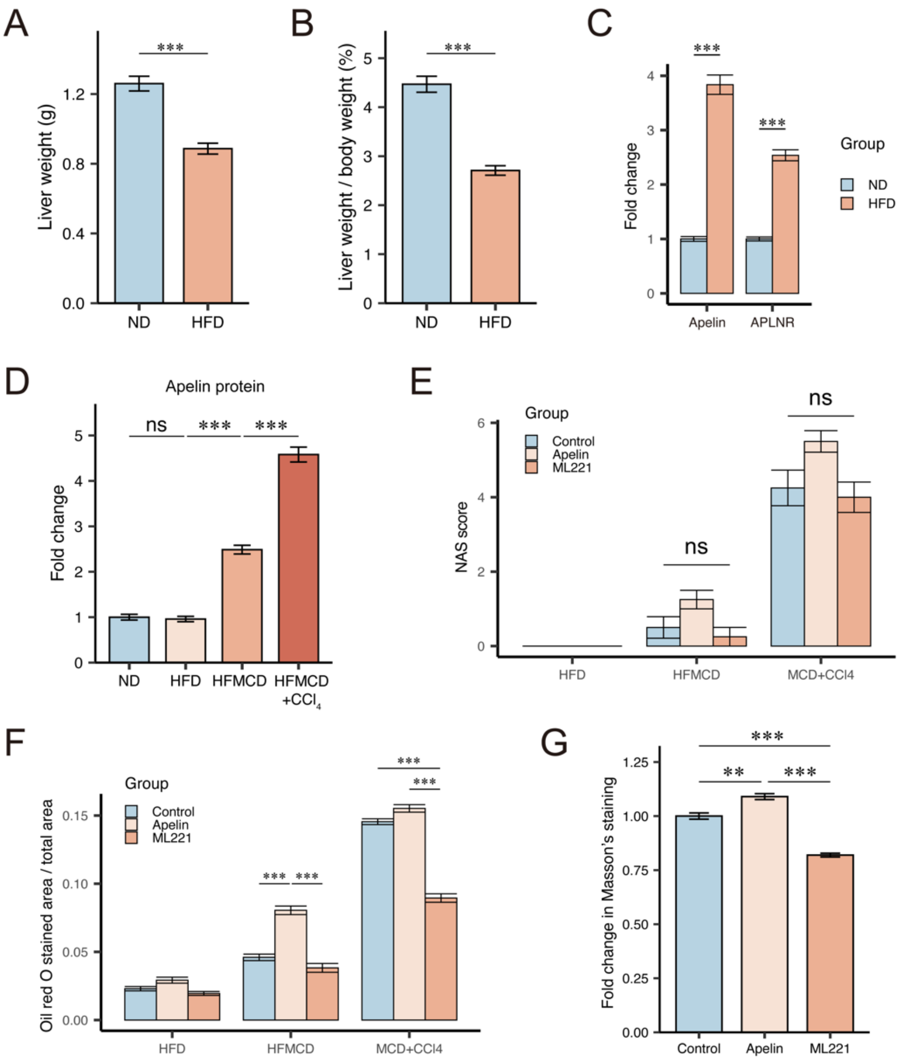

Fig. S1. Quantification of changes in mouse livers. (A-B) Liver weight of C57/BL6 mice on ND diet for 15 weeks and HFD diet for 15 weeks (n=6). (C) Protein levels of apelin and APLNR in ND and HFD mouse livers were quantified by Western blot (n=6). (D) Protein levels of apelin in different groups of mouse livers were quantified by Western blot (n=4). (E-G) NAS score, quantification of Oil red O stain and Masson stain in different groups of mouse livers (n=4). The P values are calculated by two-tailed unpaired Student's t-test (A-B), two-tailed unpaired Welch's t-test (C), or one-way ANOVA followed by Tukey's multiple comparisons tests (D-G). **P* < 0.05; ***P* < 0.01; ****P* < 0.001; ns represents no significance

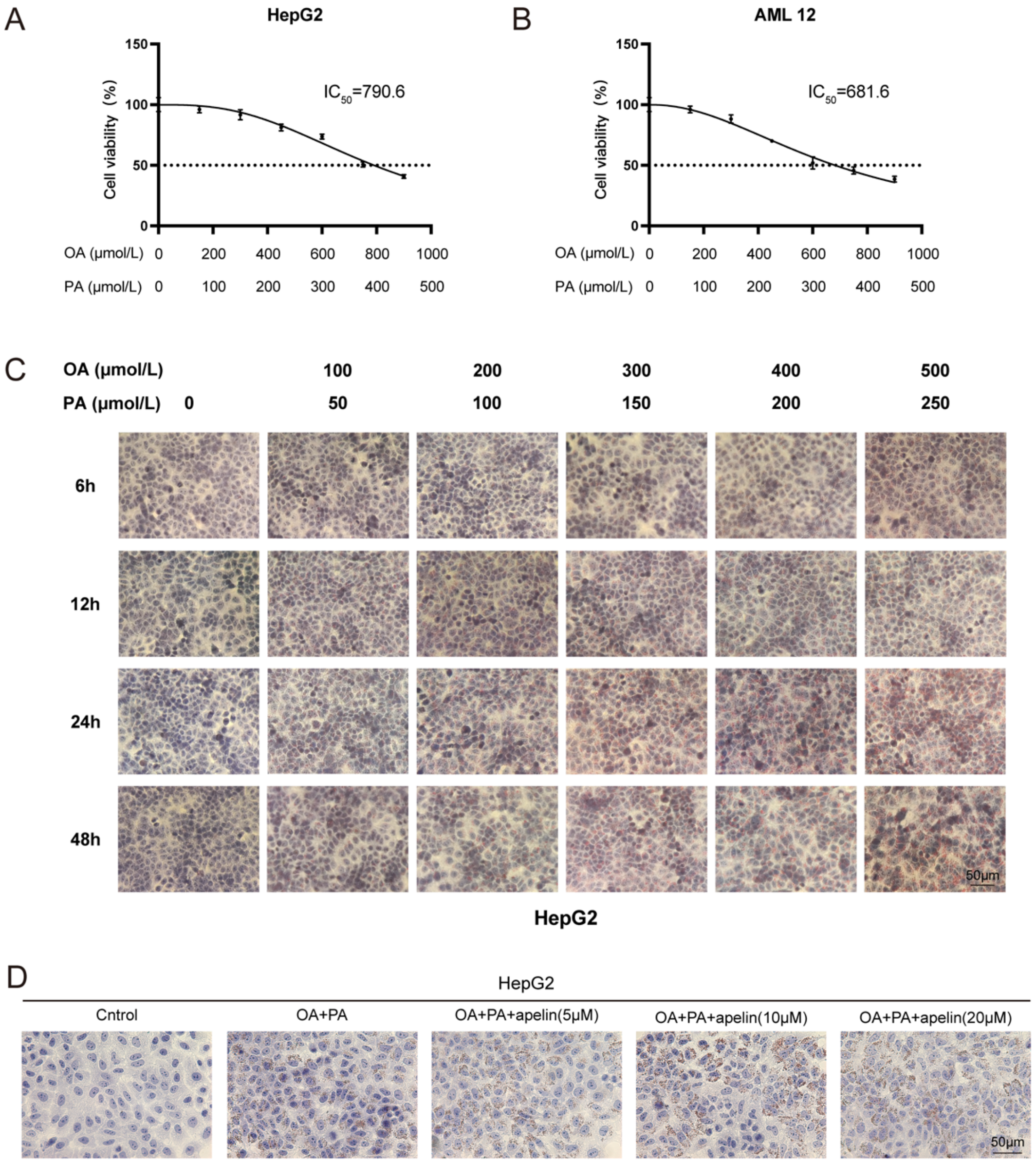

Fig. S2. Lipid accumulation models were constructed in HepG2 and AML 12 hepatocytes. (A-B) The cell viability of HepG2 cells and AML 12 cells was measured using the CCK8 assay under different concentrations of OA and PA, with the IC_{50} shown in the graph. (C) HepG2 cells were cultured with gradient concentrations of OA and PA for various durations. (D) In HepG2 cells, OA (300 μmol/L) and PA (150 μmol/L) were added to the culture medium to simulate lipid metabolism disorder in hepatocytes. Different concentrations of apelin protein were then added, and lipid droplets were observed using Oil Red O staining.

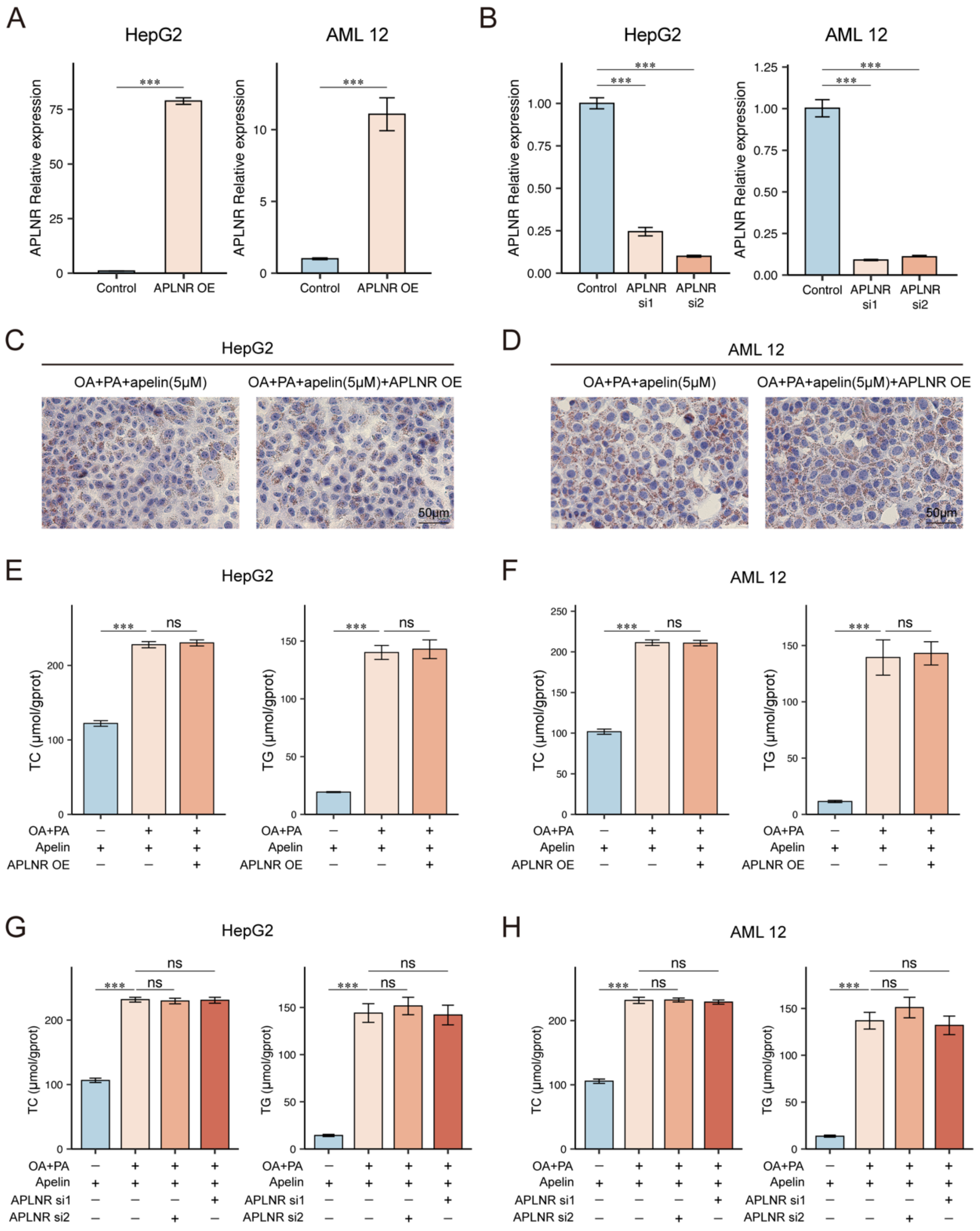

Fig.S3. Effect of Apelin and APLNR on Lipid Accumulation in Hepatocytes Induced by OA and PA. (A) Overexpression of *APLNR* in hepatocytes was validated by measuring *APLNR* mRNA using RT-PCR after plasmid transfection (n=3). (B) Interference of *APLNR* in hepatocytes was validated by measuring *APLNR* mRNA using RT-PCR after *APLNR* interference (n=3). (C-D) HepG2 and AML 12 cells were transfected with *APLNR* overexpression or control plasmids. OA, PA, and apelin were

added to the culture medium, followed by Oil Red O staining, (E-F) and measurement of intracellular TC and TG concentrations using a kit (n=12). (G-H) Compared to the control group, intracellular TC and TG concentrations were measured after *APLNR* interference (n=12). The P values are calculated by two-tailed unpaired Welch's t-test (A), or one-way ANOVA followed by Tukey's multiple comparisons tests (B, E-H). *P < 0.05; **P < 0.01; ***P < 0.001; ns represents no significance

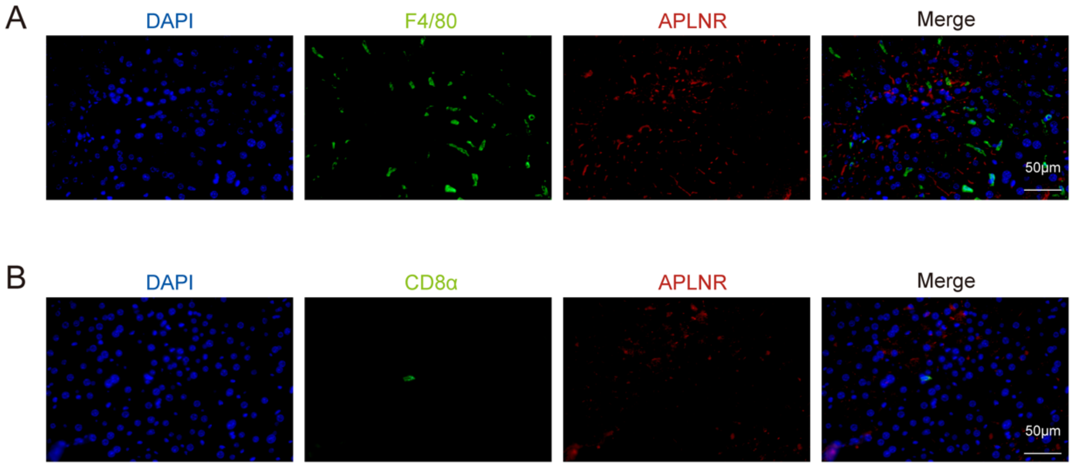

Fig. S4. Expression of APLNR on other immune cells in the liver. (A) A Immunofluorescence detection of F4/80 and APLNR in livers of mice fed with HFD and ND for 15 weeks. (B) An Immunofluorescence detection of CD8α and APLNR in livers of mice fed with HFD and ND for 15 weeks.

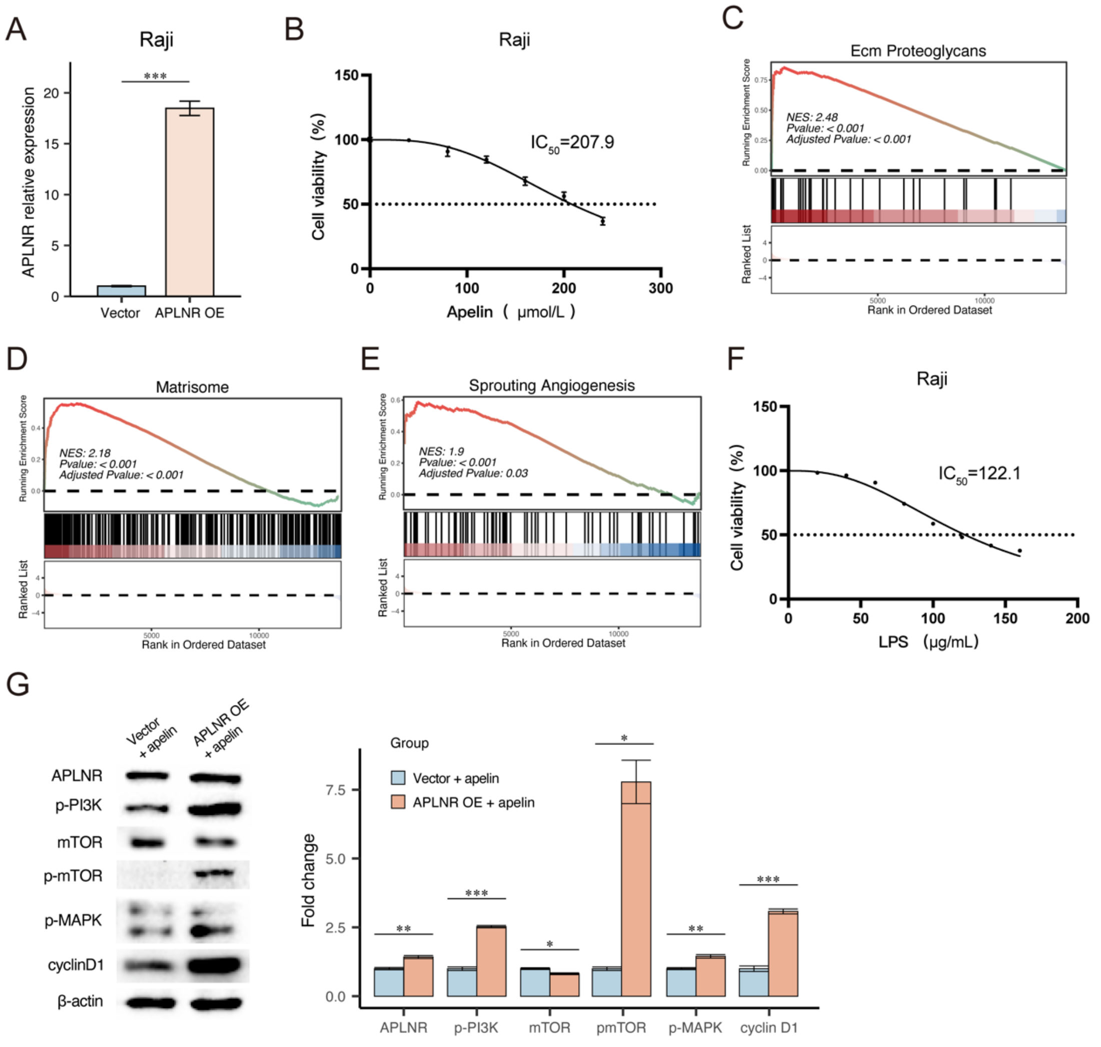

Fig. S5. The Role of Apelin and APLNR in Raji Cells. (A) After overexpressing APLNR in Raji cells using plasmid transfection, the *APLNR* mRNA levels were measured by RT-PCR (n=3). (B) The IC_{50} of apelin in Raji cells was determined. (C-E) GSEA analysis of sequencing results in Raji cells overexpressing APLNR. (F) The IC_{50} of LPS in Raji cells was determined. (G) After overexpressing APLNR in Raji cells and adding apelin, the protein levels of downstream pathway components were measured by Western blot. Quantitative results are shown on the right (n=3). The P values are calculated by two-tailed unpaired Student's t-test (A, G). *P < 0.05; **P < 0.01; ***P < 0.001

Animal Experiments

Establishment of Mouse Models for Different Stages of MASLD: To gain a more comprehensive understanding of the progression of MASLD, we developed stage-specific models using male C57BL/6 mice and implemented various dietary interventions. These included a 9-week high-fat diet with 60% of calories from fat, a 9-week methionine- and choline-deficient high-fat diet, and the same deficient diet combined with low-dose CCl_4 treatment during the final 2 weeks. Mice on a normal diet served as the control. From the 7th to the 9th week, we gave daily intraperitoneal injections of apelin, ML221, or saline to the respective groups. Throughout the study, we monitored body weight, blood glucose, lipid profiles, and liver histopathology to ensure the successful establishment of these models. At the end of the 9-week period, we collected blood and liver tissue samples for further analysis.

Plasmid transfection

According to the manufacturer's instructions, 1×10^5 cells of HepG2 and AML 12 cells were inoculated in a six-well plate. After 24 hours, the plasmid was transfected into cells with Lipofectamine 2000 (Biosharp, cat. no. BL623A). After 24 hours of transfection, cells were collected to extract total RNA for RT-PCR analysis. After 48 hours of transfection, cells were collected to extract total protein for Western blot analysis.

RNA-seq and bioinformatic analysis

A total amount of ≥1µg RNA per sample was used as the starting RNA for library construction. equencing libraries were generated using Hieff NGS Ultima Dual-mode mRNA Library Prep Kit for Illumina (Yeasen Biotechnology (Shanghai) Co., Ltd.). The libraries were sequenced on an Illumina NovaSeq platform to generate 150 bp paired-end reads, according to the manufacturer’s instructions.

Masson’s trichrome staining

A Masson’s trichrome staining kit (Solarbio, cat. no. G1340) was used according to the manufacturer’s instructions. Briefly, paraffin sections are de-waxed to water, then stained with Ponceau S solution for 5 minutes. After washing with a weak acid solution, the sections are stained with aniline blue for 2 minutes. They are then quickly dehydrated, cleared in xylene, and mounted with neutral gum.

Western blot

About 1×10^6 cells were collected and lysed with protease inhibitor-containing RIPA buffer on ice for 30 min to obtain total cellular protein extracts, which were transferred to PVDF membranes using 10% SDS–PAGE run at 90 V for 20 min and 110 V for 70 min and transferred to PVDF membranes at 350 mA. The membrane was blocked with 5% skim milk for 2 h. The corresponding primary antibodies were incubated overnight at 4°C. The protein bands were visualized with matching horseradish peroxidase-coupled secondary antibodies in the presence of an enhanced chemiluminescence (ECL) kit. The corresponding antibodies are listed in Table S1.

Quantitative real-time PCR

Total RNA was extracted from 1×10^5 cells using TRIzol reagent according to the manufacturer's instructions. Reverse transcription reagent was used to reverse transcribe the sample into cDNA, and a reaction system was conFigd to measure gene

expression by qPCR.

Immunofluorescence

A quadruple-fluorescence immunohistochemical mouse/rabbit kit (Immunoway, cat. no. RS0037) was used according to the manufacturer's instructions. After dewaxing and antigen retrieval, apply reagent B (peroxidase blocking buffer) to the paraffin sections and incubate for 15 minutes at room temperature. Following PBST washes, add the primary antibody to cover the tissue, then incubate overnight at 4°C, followed by 30 minutes at 37°C. After washing with PBST, apply reagent C (HRP polymer anti-rabbit/mouse secondary antibody) and incubate for 30 minutes at room temperature. Wash with PBST, then apply reagent D-594 (fluorescent dye), incubate for 10 minutes, and wash again. Place the slide in a container with reagent F and heat above 95°C for 15 minutes. Repeat the process for the second primary antibody using reagent D-488 as the fluorescent dye. Finally, add reagent G (containing DAPI) and seal the slide.

Immunohistochemistry

The tissue sections were heated to 80°C for a 30-minute incubation, followed by deparaffinization using xylene and absolute ethanol. After antigen retrieval, the sections were treated with the appropriate primary antibody and left to incubate overnight at 4°C. On the following day, after applying the corresponding secondary antibody, the sections underwent hematoxylin and DAB staining, and were finally sealed with a neutral resin. Staining assessment was based on the intensity of the color and the spread of stained cells. The scoring criteria were: intensity rated from 0 to 3, and the percentage of stained cells from 0 to 4, with the final score being the multiplication of both values. Two pathologists independently evaluated each section, and the mean of their scores was used for the analysis.

Hematoxylin–eosin staining (H&E)

The mouse slices were baked at 80°C for 30 minutes, dewaxed with xylene and absolute ethanol, stained with hematoxylin (4min) and eosin (1min), and finally sealed with neutral resin.

Supporting Table S1 Reagents used in the study

Reagents	Companies	Catalog#	Application
Apelin antibody	AiFang	AF06605	WB, IHC
APLNR antibody	Proteintech	#20341-1-AP	WB, IHC, IF, FCM
GAPDH antibody	Proteintech	#5	WB
CD19 antibody	Proteintech	#27949-1-AP	IF
CD19 antibody	ABclonal	A22816	FCM
p-PI3K antibody	MedChemExpress	HY-P81211	WB
p-MAPK antibody	CST	#4370	WB
mTOR antibody	Servicebio	GB11405	WB
p-mTOR antibody	Proteintech	#67778-1-Ig	WB
CyclinD1 antibody	Abcam	ab134175	WB
β-actin antibody	Servicebio	GB15001	WB
F4/80 antibody	CST	#70076	IF
CD8α antibody	CST	#98941	IF
Apelin-13	MedChemExpress	HY-P1944	Cell and animal experiments

Supporting Table S2 Primer sequence

Target	Primer sequence
APLNR	F: CTACACGTACCGGGACTATGA
	R: ACATGTTGACGAAGATGAGGTA
APLN	F: GTCTCCTCCATAGATTGGTCTGC
	R: GGAATCATCCAAACTACAGCCAG
Aplnr	F: GGTTACAACTACTATGGGGCTGA
	R: AGCTGAGCGTCTCTTTTCGC
Apln	F: TAGCCCCTGACACTGGTTGTC
	R: TTCTCCATCCCCCAAAAGC
AHNAK	F: TACCCTTCCTAAGGCTGACATT
	R: TTGGACCCTTGAGTTTTGCAT
COL6A3	F: ATGAGGAAACATCGGCACTTG
	R: GGGCATGAGTTGTAGGAAAGC
LAMB1	F: AGGAACCCGAGTTCAGCTAC
	R: CACGTCGAGGTCACCGAAAG
IRF9	F: GCCCTACAAGGTGTATCAGTTG
	R: TGCTGTCGCTTTGATGGTACT
IL10	F: GACTTTAAGGGTTACCTGGGTTG
	R: TCACATGCGCCTTGATGTCTG
CD86	F: CTGCTCATCTATACACGGTTACC
	R: GGAAACGTCGTACAGTTCTGTG
RFX2	F: AGCACAAGCTAGACCCAGTGA
	R: GGCTTCAGACGAATCCCATAG
GAPDH	F: GCATTGCCCTCAACGACCAC
	R: CCACCACCCTGTTGCTGTAG
Gapdh	F: AGGTCGGTGTGAACGGATTTG
	R: TGTAGACCATGTAGTTGAGGTCA
